# Feasibility and safety of laparoscopic pyloromyotomy using conventional instruments for infantile hypertrophic pyloric stenosis: a retrospective analysis

**DOI:** 10.3389/fped.2026.1855195

**Published:** 2026-07-28

**Authors:** Lirong Zhang, Peng Song, Yunfeng Xiao, Quankun Zhou, Jian Yang, Bo Xiang, Zhoujian Yang

**Affiliations:** 1 Mianyang Central Hospital, School of Medicine, University of Electronic Science and Technology of China, Mianyang, China; 2 Department of Pediatric Surgery, West China Hospital, Sichuan University, Chengdu, China

**Keywords:** conventional instruments, hypertrophic pyloric stenosis, infant, laparoscopic pyloromyotomy, minimally invasive surgery

## Abstract

**Background:**

This study assesses the clinical effectiveness and technical expertise associated with laparoscopic pyloromyotomy (LP) carried out using conventional instruments for the treatment of congenital hypertrophic pyloric stenosis (CHPS) in infants.

**Methods:**

We carried out a retrospective analysis of 24 patients who were diagnosed with CHPS at the Department of Pediatric Surgery, Mianyang Central Hospital, from March 2022 to December 2025. The cohort consisted of 19 boys and 5 girls, having a median age of 41 days (range: 13–123 days) and a mean weight of 3.83 ± 0.75 kg (range: 2.1–5.0 kg). All patients presented non-bilious vomiting as the primary symptom. Every patient underwent laparoscopic pyloromyotomy using conventional laparoscopic instruments.

**Results:**

Surgical procedures were successfully accomplished in all 24 cases, without the need for conversion to open surgery. The median operative time was 30 min (range: 13–60 min). All patients resumed oral feeding on the first postoperative day. Three patients (12.5%) suffered from mild and transient vomiting, which lasted for 2 days, 3 days, and 1 week respectively. These symptoms resolved spontaneously after conservative management, specifically by reducing the feeding volume and increasing the feeding frequency, without the necessity of further intervention. No other complications were detected. The postoperative hospital stay length ranged from 1 to 5 days. The follow - up durations varied from 2 months to 3 years and 11 months, during which all patients showed symptom resolution and normal growth patterns.

**Conclusion:**

Laparoscopic pyloromyotomy performed with conventional instruments is a safe and effective intervention for infants suffering from hypertrophic pyloric stenosis. This technique presents the advantages of minimal invasiveness, procedural simplicity, and low complication rates, thus standing as a viable option for extensive clinical application.

## 1 Introduction

Hypertrophic pyloric stenosis (HPS) is a leading cause of gastrointestinal obstruction in infancy. The clinical presentation typically involves progressive non-bilious vomiting, dehydration, and metabolic disturbances, necessitating timely surgical intervention. Pyloromyotomy remains the definitive treatment for HPS (1–3). In recent years, laparoscopic pyloromyotomy (LP) has gained widespread acceptance as the standard of care, favored for its minimally invasive nature, rapid recovery, and superior cosmetic outcomes. Multiple systematic reviews have demonstrated that compared with traditional open pyloromyotomy (OP), LP facilitates faster recovery of full enteral feeding and reduces the risk of surgical site infections (3–5).

Despite these advantages, LP typically requires specialized instrumentation, such as a dedicated pyloric scalpel. This dependency often limits the adoption of the technique in resource-limited settings. While alternative methods—such as umbilical extrusion of the pylorus or the use of modified, self-made instruments—have been described, their utility is often restricted by technical complexity, a steep learning curve, or concerns regarding instrument stability (6, 7). Furthermore, existing literature indicates a relatively high risk of incomplete myotomy following LP, a complication potentially associated with the laparoscopic learning curve and the precision of the instruments employed (8).

Consequently, establishing a standardized protocol for performing LP using only conventional laparoscopic instruments—such as electrocautery hooks and dissection forceps—is of significant clinical value. Such a protocol could enhance the accessibility and safety of this procedure in primary care centers and institutions with limited equipment. This study aims to summarize a single-center experience with LP performed using conventional instruments, evaluating its technical feasibility, perioperative safety, and short-term efficacy.

## 2 Materials and methods

### 2.1 Patients

We retrospectively analyzed the clinical data of 24 infants with HPS admitted to our hospital between March 2022 and December 2025.

**Inclusion criteria** were as follows: (1) infants under one year of age presenting with clinical characteristics consistent with HPS onset; (2) recurrent non-bilious vomiting confirmed by abdominal ultrasound (pyloric muscle thickness ≥4 mm, pyloric canal length ≥16 mm) (9); (3) no contraindications to general anesthesia or laparoscopic surgery; and (4) availability of complete clinical and follow-up data.

**Exclusion criteria** were: (1) presence of severe congenital malformations; (2) history of abdominal surgery; (3) inability to tolerate laparoscopic surgery due to preoperative complications such as severe dehydration, refractory electrolyte disturbances, or sepsis; and (4) loss to follow-up.

This study received approval from the Ethics Committee of our hospital (Approval No.: [S20260378-01]) and was carried out in strict accordance with the Declaration of Helsinki.

### 2.2 Preoperative evaluation and preparation

All patients underwent a physical examination, complete blood count, electrolyte panel, liver and kidney function tests, and electrocardiography prior to surgery. Abdominal ultrasound was performed by two independent sonographers to confirm the diagnosis and evaluate the degree of pyloric muscle hypertrophy. Patients presenting with electrolyte disturbances received intravenous fluid resuscitation to correct levels to the normal range and ensure hemodynamic stability before surgery. A gastric tube was inserted preoperatively to decompress the stomach, thereby reducing the risk of aspiration during anesthesia and improving surgical field exposure.

### 2.3 Surgical methods

**Figure 1:**
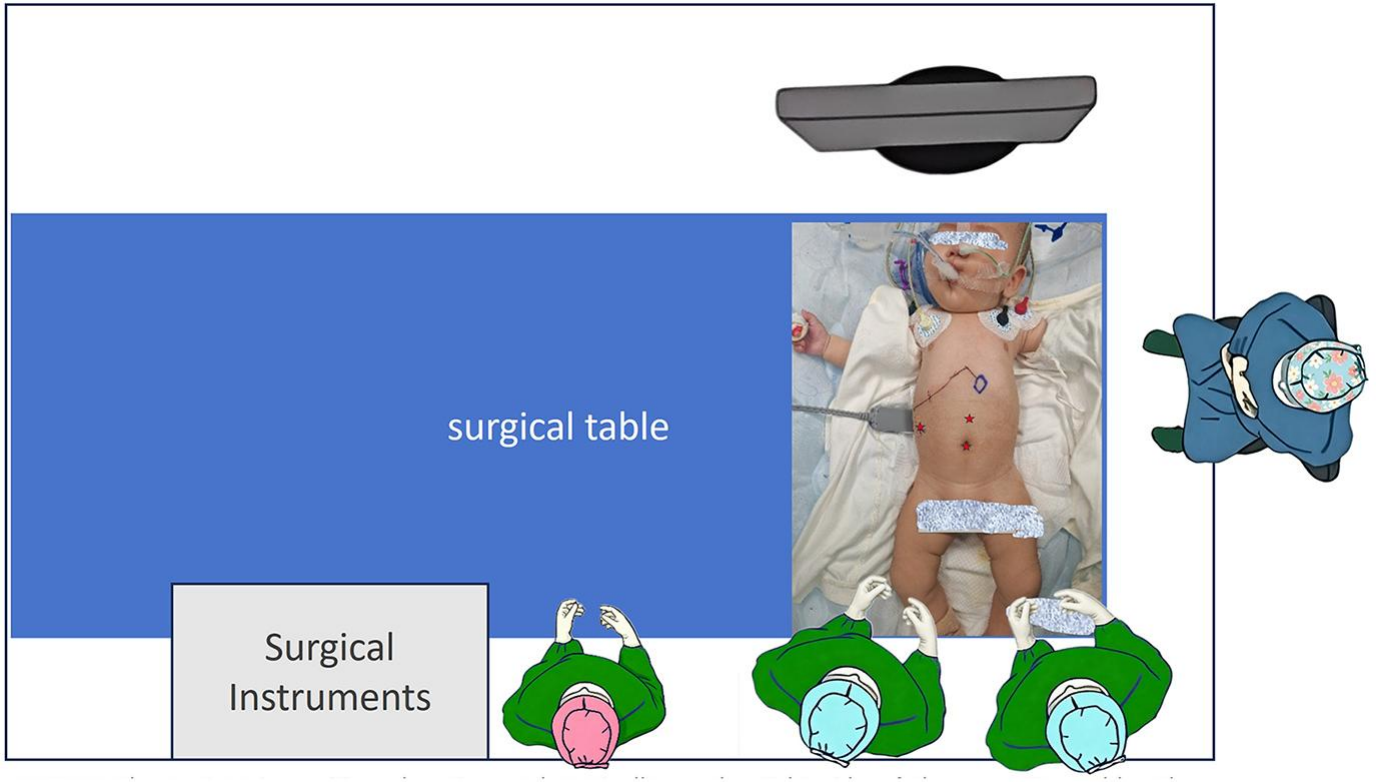
The patient is positioned supine and vertically on the right side of the operating table. The monitor is placed at the head of the bed. The surgeon stands at the lower-left position relative to the patient, and the cameraman stands at the foot of the bed. The red five-pointed star marks the positions of the surgical ports.

**Figure 2:**
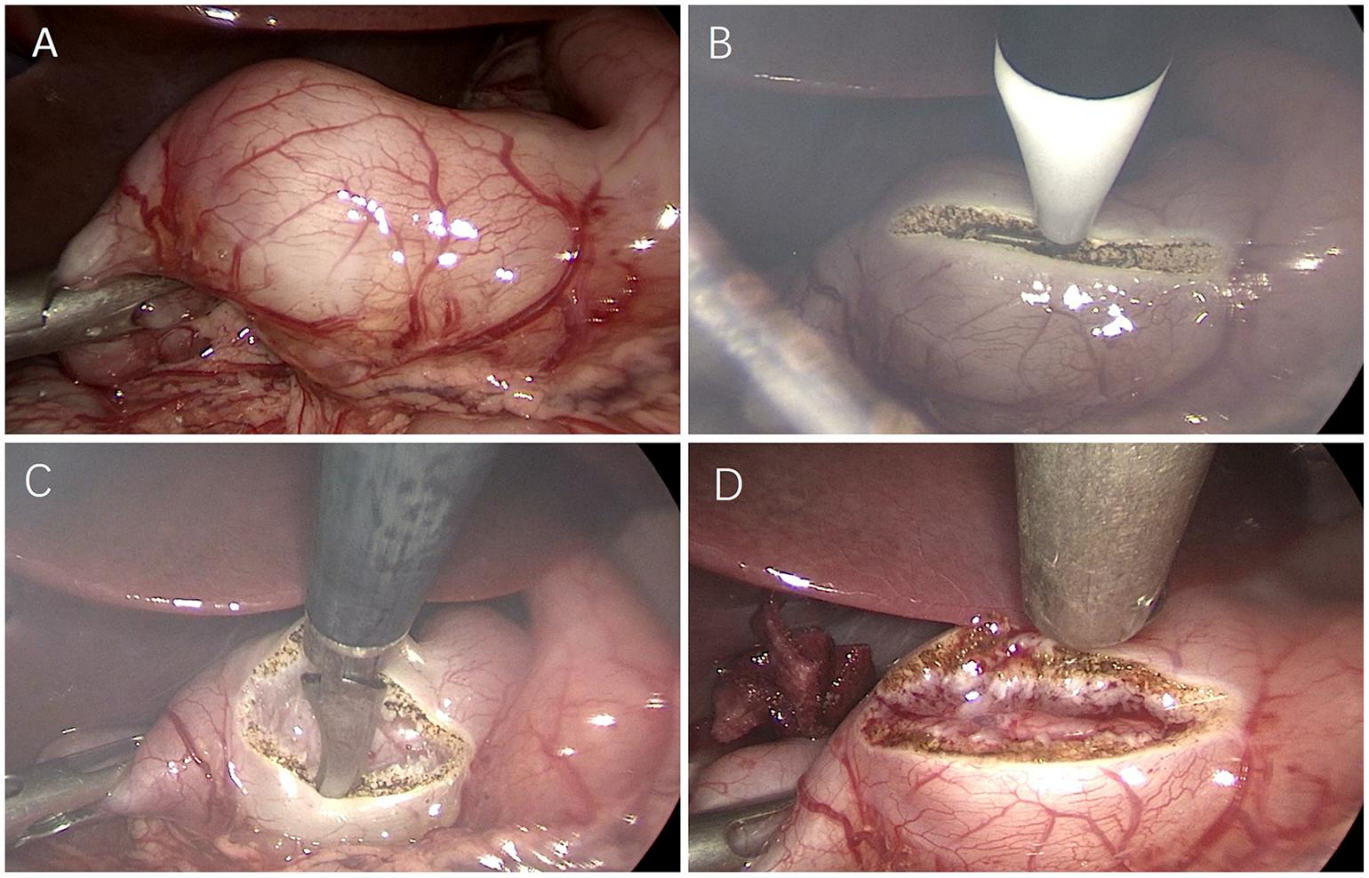
The image illustrates the surgical procedures of laparoscopic pyloromyotomy employing conventional instruments: the surgeon firmly grasps a long atraumatic forceps with the left hand to clamp and stabilize the hypertrophic pyloric canal. Subsequently, the surgeon rotates it appropriately to expose the avascular area along the antimesenteric border of the pylorus, thus fully visualizing the operative field **(A)**. The intended incision line is precisely demarcated with electrocautery. The serosa and muscular layer at the center of the pyloric canal are incised meticulously along the demarcation. The depth of the incision is carefully regulated to just accommodate the tip of the electrocautery hook. Then, the incision is extended to both ends to divide the partial muscular layer **(B)**. A laparoscopic curved forceps is smoothly inserted into the middle of the pyloric canal. The hypertrophic muscular layer is gently dissected along the incision until the mucosa is completely everted **(C)**. Hemostasis is effectively accomplished by compression with gauze or cotton pads. Electrocoagulation is avoided to prevent thermal damage to the mucosa. The integrity of the mucosa is thoroughly examined, and perforation is ruled out by insufflating the gastric cavity through a nasogastric tube **(D)**.

All procedures were performed under general anesthesia with endotracheal intubation. The patient was placed in a supine position on the operating table. The monitor was positioned at the head of the bed, the primary surgeon stood at the patient's lower left, and the camera operator stood at the foot of the bed (Figure 1). The specific surgical steps were as follows:

1. **Establishment of Pneumoperitoneum:** A 5-mm trocar was inserted via an umbilical puncture. Pneumoperitoneum was established with an abdominal pressure maintained at 6–8 mmHg and a gas flow rate of 0.5–1.0 L/min. A 5-mm laparoscope was inserted to explore the abdominal cavity.
2. **Placement of Working Ports:** Under direct visualization, two 5-mm trocars were inserted into the right upper abdomen and the upper mid-abdomen to serve as working ports (Figure 1).
3. **Myotomy Incision:** After the surgical field is completely exposed (Figure 2A), the surgeon switched the right-hand instrument to an electrocautery hook. The anticipated incision line was first cauterized to mark the serosa. The serosal and muscular layers were then incised along this mark at the central portion of the pyloric canal in electrocautery mode, with the energy output controlled at 15–20. The incision depth was controlled to approximate the depth of the cautery hook tip (2.5 mm) ensuring the muscular layer was cut through to both ends (Figure 2B).
4. **Separation of the Muscular Layer:** The electrocautery hook was replaced with a laparoscopic grasping forceps (or curved dissector). The instrument was inserted into the myotomy incision, and the hypertrophic muscle fibers were gently separated along the incision until the underlying mucosal layer bulged completely (Figure 2C).
5. **Hemostasis and Mucosal Integrity:** Minor intraoperative bleeding was managed by gauze compression; electrocautery hemostasis was avoided to prevent thermal injury to the mucosa. Air was insufflated via the gastric tube to distend the stomach and check for mucosal integrity, thereby excluding perforation (Figure 2D). No abdominal drain was placed.

### 2.4 Postoperative management

Postoperative monitoring included vital signs, abdominal examination, and assessment of gastrointestinal function recovery. Prophylactic antibiotics were administered for 24 to 48 h. Oral feeding was initiated on the first postoperative day, starting with small volumes of breast milk or formula, which were gradually increased as volume was advanced. Patients were discharged upon meeting the following criteria: stable vital signs, absence of vomiting (or only mild transient vomiting), tolerance of normal feeding, and passage of stool.

### 2.5 Outcome measures

**Primary outcome measures** included surgical success rate, operative time (defined from establishment of pneumoperitoneum to skin closure), duration of postoperative vomiting, length of postoperative hospital stay, and incidence of complications (wound infection, pneumonia, mucosal perforation, recurrence, etc.).

**Secondary outcome measures** included postoperative growth and cosmetic satisfaction of the abdominal wall, as evaluated during follow-up.

### 2.6 Follow-up

The follow-up period ranged from 2 months to 47 months, with a median duration of 2 years and 2 months. During follow-up, physical examinations and abdominal ultrasounds were performed to assess pyloric patency, disease recurrence, and growth and development status.

### 2.7 Statistical analysis

Data were analyzed using SPSS version 26.0 software. Normally distributed continuous data were expressed as mean ± standard deviation $\left(\bar{x}\pm s\right)$. Skewed continuous data were expressed as median (range). Categorical data were expressed as number (percentage).

## 3 Results

### 3.1 Baseline characteristics

The study cohort comprised 24 infants with HPS, including 19 boys (79.2%) and 5 girls (20.8%). The median age was 41 days (range: 13–123 days), and the mean weight was 3.83 ± 0.75 kg (range: 2.1–5.0 kg). All patients presented with recurrent non-bilious vomiting, with a median symptom duration of 9 days (range: 1–60 days).

Four patients presented with concurrent congenital anomalies: one case of Esophageal hiatal hernia (type I) (4.2%), one case of ventricular septal defect (4.2%), and two cases of indirect inguinal hernia (8.3%). The remaining patients had no other apparent congenital malformations (Table 1).

**Table 1:** Baseline characteristics and clinical outcomes of 24 infants with hypertrophic pyloric stenosis.

Variable	Value
Total cases (Male/Female), *n*	24 (19/5)
Age, days	Median (range): 41 (13–123)
Body weight, kg	Mean ± SD: 3.83 ± 0.75 (range: 2.1–5.0)
Duration of vomiting, days	Median (range): 9 (1–60)
Associated malformations, *n* (%)	4 (16.7)
Esophageal hiatal hernia	1 (4.2)
Ventricular septal defect	1 (4.2)
Indirect inguinal hernia	2 (8.3)
Operative time, minutes	Median (range): 30 (13–60)
Postoperative hospital stay, days	1–5
Postoperative complications, *n* (%)	
Vomiting	3 (12.5)
Wound infection	0
Mucosal perforation	0
Follow-up	
Duration(months), median (range)	35 (2–47)
Recurrent vomiting, *n*	3
Readmission, *n*	0
Reoperation, *n*	0

### 3.2 Surgical outcomes

All procedures were performed by board-certified pediatric surgeons holding senior academic titles (Associate Professor or above), none of whom had prior experience with dedicated pyloromyotomy instruments.All 24 procedures were successfully completed laparoscopically, with no conversions to open surgery required. The median operative time was 30 min (range: 13–60 min). Three patients (12.5%) experienced mild, transient vomiting postoperatively, with durations of 2 days, 3 days, and 1 week, respectively. Symptoms resolved spontaneously following dietary modification (reduced feeding volume and increased frequency); no additional medical intervention was required. All patients resumed oral feeding on the first postoperative day. The length of postoperative hospital stay ranged from 1 to 5 days.

### 3.3 Follow-up results

**Figure 3:**
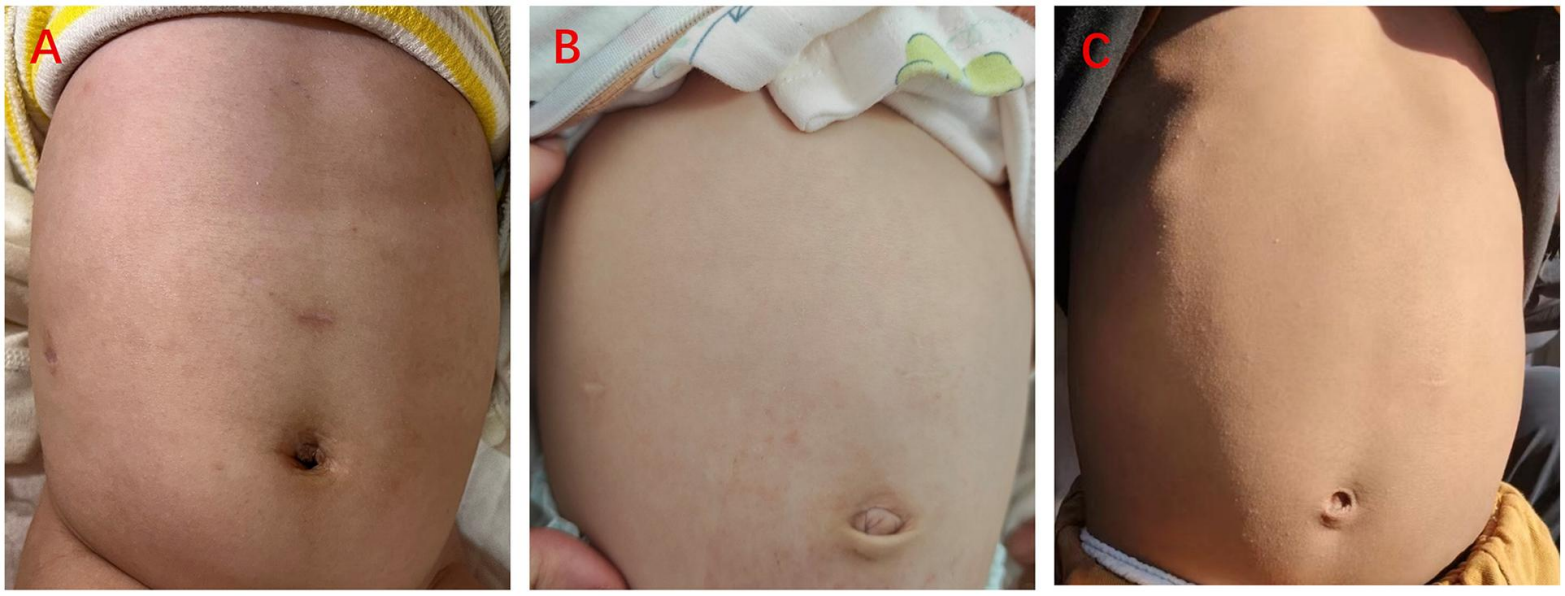
The appearance of the abdomen post-operatively—One month after surgery **(A)**; Six months after surgery **(B)**; thirteen months after surgery **(C)**.

During the follow-up period, vomiting resolved completely in all patients, and abdominal ultrasound examinations confirmed no recurrence of HPS. All patients exhibited normal growth and development. Abdominal incisions healed well with minimal scarring, yielding satisfactory cosmetic outcomes (Figure 3).

No postoperative complications were observed, including wound infection, pneumonia, mucosal perforation, readmission, reoperation, or infections at other sites.

## 4 Discussion

Although the incidence of hypertrophic pyloric stenosis (HPS) is relatively low, it represents a major surgical emergency in the neonatal period. Failure to treat HPS in a timely manner can lead to serious complications (8). While conservative management is an option, it necessitates long-term parenteral nutritional support, its efficacy remains uncertain, and the need for repeated invasive procedures is often poorly accepted by families (10). Consequently, surgical intervention remains the gold standard treatment for HPS.

Alain et al. first reported the successful application of laparoscopic pyloromyotomy (LP) in 1990. However, due to the fragility of the pyloric tissues, this technique carries an inherent risk of mucosal perforation or incomplete myotomy (11). To address these technical challenges, Najmaldin et al. subsequently detailed the use of dedicated dissecting knives and forceps, which mitigated some instrumental limitations (12). Later, Akl et al. compared LP with open surgery, finding no significant differences in operative time, length of hospital stay, or complication rates between the two approaches. Nevertheless, LP has become the standard of care due to its superior cosmetic outcomes, faster time to resumption of feeding, and favorable long-term profile (8).

Despite these advantages, the widespread adoption of laparoscopic techniques remains restricted in clinical practice. Barriers include geographic limitations, the low incidence of the disease, a lack of specialized equipment in many institutions, and limited availability of centers offering this procedure (13–16). In this study, we utilized conventional laparoscopic instruments—tailored to local anatomical characteristics—to streamline the surgical workflow. This approach reduces the technical difficulty of the procedure and offers a feasible minimally invasive treatment option for resource-limited settings.

### 4.1 Clinical outcomes and technical feasibility

All patients in this cohort successfully completed the procedure without conversion to open surgery, intraoperative perforation, or other serious complications. There were no unplanned readmissions, confirming the safety and feasibility of the technique. Based on our data, we attribute the success of the operation to adherence to standardized procedures and precise intraoperative technique. We summarize the core technical points of laparoscopic pyloromyotomy using conventional instruments as follows:

1. **Optimized Patient Positioning** Our positioning strategy differs slightly from that proposed by Chiarenza (10). The patient is placed laterally on the operating table, with the surgeon standing at the patient's lower left and the camera operator at the foot of the bed. This arrangement conforms to the ergonomic practices of the surgical team and facilitates the manipulation of conventional instruments within the limited space of the infant's abdominal cavity (Figure 1).
2. **Adequate Exposure of the Surgical Field** Gastric contents are emptied via a gastric tube preoperatively. If emptying is suboptimal, the tube position can be adjusted under direct laparoscopic vision. This step not only reduces the risk of aspiration during anesthesia but also prevents gastric distension from occupying the surgical space, thereby improving exposure. A non-traumatic grasper is used in the left hand to stabilize the pyloric canal; gentle rotation is applied to fully expose the avascular zone on the contralateral mesenteric border. This technique minimizes intraoperative bleeding and enhances the clarity of the surgical field (Figure 2).
3. **Strategic Port Placement** The layout of the working ports is critical. The left port (right upper abdomen) is positioned slightly lower to facilitate stable fixation of the pyloric canal. Conversely, the right port (upper mid-abdomen) is placed closer to the pylorus, allowing for direct incision and dissection, thereby reducing the technical challenge associated with conventional instruments (Figure 1).
4. **Precise Incision of the Pyloric Muscle** Incising the pyloric muscle layer is the core step of the operation. Some studies suggest that LP carries a higher risk of incomplete incision and reoperation due to limited visualization, leading surgeons to adopt a conservative incision range to avoid perforation (17). While maintaining a 2 cm incision length is suggested to ensure completeness, this still carries perforation risks (18). We have refined the incision technique based on the following principles:
  - **Site Selection:** The incision is placed at the antimesenteric border, an area characterized by fewer blood vessels, greater tissue thickness, and a lower risk of bleeding and perforation (19).
  - **Length Determination:** Incision length is individualized based on preoperative ultrasound measurements, intraoperative direct observation, and tactile feedback via the instruments. Discriminating tissue texture is vital: the normal intestine is soft and easily grasped, whereas the thickened pylorus is hard and tends to slip when clamped (Figure 2A).
  - **Technique:** The central portion of the pyloric canal serves as the primary safe zone for incision. The cuts at both ends of the canal are made shallower but must penetrate the muscular layer to ensure smooth separation. Failure to fully transect the muscle prior to dissection can cause local bleeding, obscure the field, and result in incomplete myotomy.
5. **Gentle Dissection and Compression Hemostasis** Dissection of the muscular layer begins at the center of the pyloric canal. Curved forceps are used to gently separate the hypertrophic muscle fibers, minimizing the risk of mucosal injury. For minor intraoperative bleeding, gauze or cotton pledgets are used for compression hemostasis. Electrocoagulation is avoided to prevent thermal injury and delayed mucosal perforation (Figure 2B). The absence of active bleeding in all patients in this study confirms the safety and efficacy of this hemostatic method.
6. **Mucosal Integrity Check** Following separation, the mucosa should bulge completely. An air insufflation test via the gastric tube is performed to verify mucosal integrity. If perforation is detected, immediate conversion to open surgery for mucosal repair is mandatory, accompanied by local drainage and prolonged postoperative fasting. No perforations occurred in this cohort.
7. **Management of Postoperative Vomiting** Mild, transient postoperative vomiting is often attributed to local edema. In our series, three patients experienced mild vomiting, which resolved successfully through dietary modification (reduced feeding volume with increased frequency).

### 4.2 Efficacy and learning curve

The median operative time was 30 min (range: 13–60 min), with no serious complications observed. During follow-up, there was no recurrence of vomiting, and ultrasound confirmed no recurrence of HPS, indicating positive long-term outcomes. These results validate the safety, efficacy, and reproducibility of this standardized procedure, particularly regarding the techniques of incision and separation.

Laparoscopic pyloromyotomy has a recognized learning curve, requiring significant surgeon experience in the early stages (20). Our center adopted a cautious approach during the initial implementation of this technique, resulting in longer operative times. However, as we mastered these technical points and workflow, the procedures became smooth and efficient. We suggest that surgeons attempting this technique should possess solid experience in both open surgery and conventional laparoscopic procedures.

### 4.3 Limitations

This study has several limitations. First, it is a single-center retrospective study with a small sample size (*n* = 24), which may limit the generalizability of the conclusions. Second, the lack of a control group (e.g., a specialized instrument group or an open surgery group) prevents direct comparison of efficacy and safety between different methods. Third, while the follow-up duration was sufficient to assess short- and medium-term outcomes, the lack of long-term data limits our ability to evaluate the impact of surgery on long-term growth and development. Future multicenter, prospective controlled studies with larger sample sizes and extended follow-up periods are warranted to further verify the efficacy and safety of this technique.

## 5 Conclusion

Laparoscopic pyloromyotomy using conventional instruments, such as the electrocautery hook and laparoscopic grasping forceps, is a safe, effective, and feasible treatment for neonatal hypertrophic pyloric stenosis. This method offers the benefits of minimal trauma, rapid postoperative recovery, and low complication rates without the need for specialized equipment. Through standardized surgical procedures and precise intraoperative technique, satisfactory clinical outcomes and cosmetic results can be achieved. This approach is particularly suitable for remote areas and medical institutions with limited resources and is recommended for broader clinical adoption.

## Data Availability

The original contributions presented in the study are included in the article/supplementary material, further inquiries can be directed to the corresponding author.
